# Infanticide; Contusion and Flattening of the Head

**Published:** 1846-06

**Authors:** 


*Infanticide; Contusion and Flattening of the Head.*—A young girl, in whom the exterior signs of advanced pregnancy had been noticed, was delivered clandestinely; being suspected of infanticide she obstinately denied both her pregnancy and accouchment. A professional man, called in to examine her, ascertained that she had been recently delivered. She then confessed that she had up to that time endeavoured to deceive, and added that while alone in her bed-room she was delivered of a dead child, which she had concealed (*enfoui*) three days afterwards in a place which she pointed out.

An inspection of the body was ordered, and the examination shewed that the child was born at full term, well formed, viable, and that it had lived. There was an evident flattening of the cranium in the transverse direction, and considerable ecchymosis, or rather sanguineous effusion in the cellular tissue, uniting the skin to the bones of the skull. This effusion principally occupied the two parietal regions, and was much less marked at the vertex and posterior part of the head. The medical men pronounced this to be the cause of the death of the infant.

It remained to inquire whether these traces of violence were the result of crime or of accident. From the account given it was possible that a compression so considerable as this might have been the result of labour, but of a labour long and difficult. Now, from the statement of the accused herself, the labour had been neither long nor difficult, and had been accomplished without assistance. It could not therefore be the labour which had caused the flattening of the cranium. Feeling the necessity of explaining this condition of the body, the accused pretended that this flattening might have been caused by the stones which she had placed upon the body in burying it. The ecchymosis, however, incontestibly proved that the injury had taken place during life, and all the circumstances seemed to establish the guilt of the accused.

It should, however, always be borne in mind that without the aid of moral proof of guilt, there is, perhaps, room for some reserve in regard to the facts elicited by the medico-legal investigation; for as the physician consulted very properly pointed out, it was not absolutely shown that the lesions revealed by the autopsy might not have been the result even of the labour. Influenced by these considerations, the Court referred to the jury the question of homicide by imprudence, which was affirmed.—*Gaz. Med. de Paris.*

[Looking to all the circumstances, there is, perhaps, little ground for question, but that in this instance the death of the infant was the result of crime. The following case, however, shows the necessity for extreme caution on the part of medical witnesses, and fully justifies the reserve manifested in the one just detailed.]—*Ed. Med. and Sur. Jour.*

